# Over-Eating

**Published:** 1858-03

**Authors:** 


### OVER-EATING.

How many people eat to make it even? All the butter is gone, but the bread is not quite eaten, so another piece of butter is taken; but it was too much, and the bread has given out!

How many a time has the reader eaten some remnant on his plate, not because he wanted it, but to prevent its being wasted! How often have you eaten as much as you wanted, and were about pushing back from the table, when very unexpectedly a new dish, or splendid-looking pudding, dumpling, or pie, is presented, and you immediately "set to," and before you are done, have eaten almost as much in bulk as you had done before!

Many a time have you gone down to the table, not only without an appetite, but with almost a feeling of aversion to food; and yet you tasted this, and that, and the other, and before you were aware of it, you had "made out" a considerable supper!

All these practices are wasteful, hurtful, and beastly—no, we recall that: we are doing Mr. Pig an injustice; for, like all other respectable animals, when he "is done," he "quits"—a thing which rational man seldom does.

